# A Brief Review of Aptamer-Based Biosensors in Recent Years

**DOI:** 10.3390/bios15020120

**Published:** 2025-02-18

**Authors:** Wenjing Wang, Yumin He, Suxiang He, Lei Deng, Hui Wang, Zhong Cao, Zemeng Feng, Benhai Xiong, Yulong Yin

**Affiliations:** 1 Key Laboratory of Agro-Ecological Processes in Subtropical Region, Institute of Subtropical Agriculture, Chinese Academy of Sciences, Changsha 410125, China; wangwenjing1020@stu.hunau.edu.cn (W.W.); heyumin@usc.edu.cn (Y.H.); hesuxaing@stu.hunau.edu.cn (S.H.); yinyulong@isa.ac.cn (Y.Y.); 2 Zhongke Jieyun (Beijing) Information Technology Co., Ltd., Beijing 101400, China; 3 Department of Biochemistry and Molecular Biology, Hengyang Medical School, University of South China, Hengyang 421001, China; 4 College of Chemistry and Materials Science, Hunan Agricultural University, Changsha 410128, China; 5 School of Computer Science and Engineering, Central South University, Changsha 410075, China; leideng@csu.edu.cn; 6 State Key Laboratory of Animal Nutrition, Institute of Animal Science, Chinese Academy of Agricultural Sciences, Beijing 100193, China; wanghui10@caas.cn (H.W.); xiongbenhai@caas.cn (B.X.); 7 Hunan Provincial Key Laboratory of Cytochemistry, School of Chemistry and Biological Engineering, Changsha University of Science and Technology, Changsha 410114, China; caoz@csust.edu.cn

**Keywords:** peptide, nucleic acid, aptamer, biosensor, review

## Abstract

Aptamers have recently become novel probes for biosensors because of their good biocompatibility, strong specificity, and high sensitivity. Biosensors based on peptides or nucleic acid aptamers are used in implantable and wearable devices owing to their ease of synthesis and economic efficiency. Simultaneously, amphoteric ionic peptides are being explored as antifouling layers for biosensors resistant to interference from extraneous proteins in serum. Thus, this paper reviews recently developed aptamer-based biosensors and introduces peptide- and nucleic acid-based biosensors, while focusing on the three primary classes of biosensors: electrochemical sensors, fluorescent or colorimetric biosensors, and electroluminescent sensors. Furthermore, we summarize their general construction strategies, describe specific electrochemical sensors that use peptides as an antipollution layer, and elucidate their advantages.

## 1. Introduction

Aptamers have recently gained significant attention as probes for clinical diagnosis, biosecurity, chemical and biological analyses, environmental control, and healthcare. Based on a summary of previous studies, aptamers are typically classified into nucleic acid and peptide aptamers. Because of their unique chain-like structure, aptamers are easily modified and can be folded into various conformations [1,2]. The conformational change that occurs when an aptamer binds to an analyte is typically determined by the aptamer sequence, initial structure, pH, temperature, and solution composition [3]. Stability, strong binding, low molecular mass, and the ease of synthesis and modification are the advantages of aptamers over antibodies or enzymes [4]. In addition, the unique natural advantages of aptamers include specificity, economic efficiency, standard synthesis protocols, accessibility, physical and chemical versatility, chemical combinations, and screening in random libraries [5].

Nucleic acid aptamers are typically single-stranded DNA and RNA molecules found in oligonucleotide libraries through the systematic evolution of ligands by exponential enrichment (SELEX) [6,7]. The interaction between the ligand and aptamer depends on the nature of the analyte, three-dimensional structure of the aptamer, and charge they carry [8,9]. The interactions between nucleic acid aptamers and analytes can be classified as electrostatic interactions, van der Waals forces, shape complementarity, superposition of flat groups, or hydrogen bonding [10,11]. The unique binding of nucleic acid aptamers to biological molecules depends on the unique arrangement of nucleotides and their three-dimensional structures.

Peptide aptamers are naturally occurring short-chain polypeptides comprising amino acids that have been screened for specific binding to biomolecules, such as cellular proteins, metal ions, proteases, kinases, bacteriophages, nucleic acids, and antibodies [12,13]. Self-assembled peptides typically bind to their targets via non-covalent intermolecular interactions, electrostatics, aromatic p-layer stacking, hydrogen bonding, hydrophobic interactions and van der Waals forces [14,15]. The 20 naturally occurring amino acids comprise numerous polypeptides that share the same basic structure and differ only in their side-chain groups. The close proximity of different side-chain groups results in a unique conformation of the polypeptide [16]. Owing to the presence of carboxyl and protonated amino groups, the peptide facilitates the formation of a hydrated membrane via hydrogen bonding, which reduces protein adsorption owing to hydrophobic interactions [17]. Electrically neutral peptides effectively prevent the adsorption of positively or negatively charged proteins [18,19]. Therefore, peptide-based aptamer sensors have attracted increasing attention.

Combining aptamers with signaling molecules to construct biosensors has seen widespread application. Because of their versatility in forming several tertiary structures, aptamers can also be used directly as probes for building sensors. The interaction between the aptamer and target can be detected using potentiometric, galvanometric, impedance, or surface plasmon resonance methods. Aptamer-based biosensors exhibit excellent performance and offer opportunities for the mass production and long-term preservation of sensors. Therefore, they have played significant roles in the development of modern biosensors.

This paper focuses on the characteristics of nucleic acid aptamers and peptide aptamers, as well as the aptamer-based sensors of recent years, and discusses the features of these sensors as well as their innovativeness. The aptamer-based sensors are categorized into three main groups: electrochemical sensors, photochemical sensors, and electroluminescent sensors. Since electrochemical and photochemical sensors are expected to realize real-time dynamic monitoring and rapid detection, this paper focuses on these two parts. At the same time, our focus is mainly on real-time rapid detection for in vivo or intracellular use in animals, and, therefore, the examples are also focused on this aspect.

## 2. Aptamer-Based Biosensors

Peptide aptamers are naturally evolving, screened, and validated short chains of amino acids with a specific sequence that recognizes its target. Various polypeptides share the same backbone, and their specific recognition function is attributed to their amino acid sequences and because different amino acids have different side chain (–R) groups. Depending on the R group, amino acids can be classified as charged, hydrophilic, or hydrophobic [20,21]. The order in which amino acids are arranged determines peptide conformation. Because of their high affinity and specificity, peptide aptamers have been used to construct electrochemical sensors for various conduction pathways. The exploration of peptide-based biosensors is increasing because of their ease of synthesis and biocompatibility compared to antibodies, which require in vivo environments and have a complex synthesis process. Polyethylene glycol (PEG) is easily oxidized under physiological conditions, and zwitterionic polymers have a complex synthesis process; therefore, using peptides avoids these disadvantages [22,23].

Unlike peptide aptamers, nucleic acid aptamers are commonly selected using the SELEX and typically comprise 20–100 nucleotide units linked via phosphate dihydrogen bonds [24]. Five basic nucleic acid aptamer units are similarly sequenced in a particular way to achieve specific recognition functions. Nucleic acid aptamers, a rapidly emerging subject, are attracting increasing interest from various fields. This is particularly true for the analytical assays and targeted therapeutics used in clinical medicine. An adaptive conformational change is often observed upon the binding of an aptamer to its target. This phenomenon renders it easy to design biosensors with specific and rapid responses in complex biological environments [25].

### 2.1. Electrochemical Sensors

**Figure 1:**
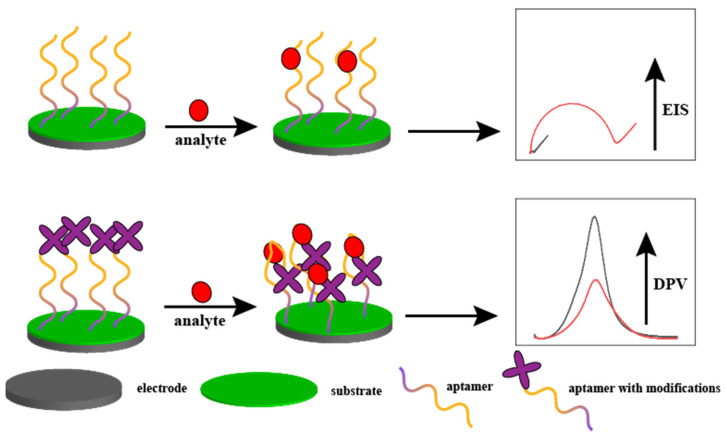
Aptamer-based detection mechanism for electrochemical sensors. The upper figure shows the electrochemical impedance method, which results in an increase in electrochemical impedance when the analyte binds to the aptamer; the lower figure shows the current method, which results in an increase in induced current when the analyte binds to the aptamer, causing the signaling molecules at the end of the aptamer to approach the electrode interface.

This section summarises the current aptamer-based electrochemical sensors and provides selected cutting-edge examples. Figure 1 illustrates the aptamer-based detection mechanism for electrochemical sensors.

The detection principles of most electrochemical sensors are divided into two parts. (1) The aptamer is fixed to the electrode surface. When an analyte is present in the solution, it is attracted to the aptamer and adsorbed onto the electrode surface. This phenomenon causes a decrease in the electron transfer efficiency of the electrode surface and an increase in resistance. The higher the concentration of the analyte, the higher the resistance, which is used as a means of detection. (2) The end of the aptamer, which is typically a signal molecule, such as ferrocene, is modified when the analyte is present in the solution. Binding of the analyte to the aptamer results in a change in the conformation of the aptamer, which changes the distance between the signaling molecule and the electrode surface. This process increases or decreases the response current. The higher the concentration of the analyte, the greater the amount of change in the response current, which can be used as a means of detection.

Chang et al. [26] constructed an impedance-based biosensor to detect L-ascorbate 6-phosphate lactonase (UlaG). Anionic and cationic materials were deposited on the carbon electrode as an antifouling layer. The antifouling performance was significantly enhanced when comparing the luminescence intensity of the chromogenic fluorescent protein before and after deposition. Subsequently, the peptide was deposited on top of the antifouling layer. When UlaG was introduced in the solution, it bound to the end of the peptide and increased the electrode surface resistance. The higher the concentration of UlaG, the higher the resistance, which was used as an electrical signal to detect UlaG. The authors creatively used amphiphilic ions as a modifying layer, which can be used both to provide electrical signals and to resist interference. The sensor had a detection range and limit of 0.25–7.5 nM and 0.5 nM, respectively.

**Figure 2:**
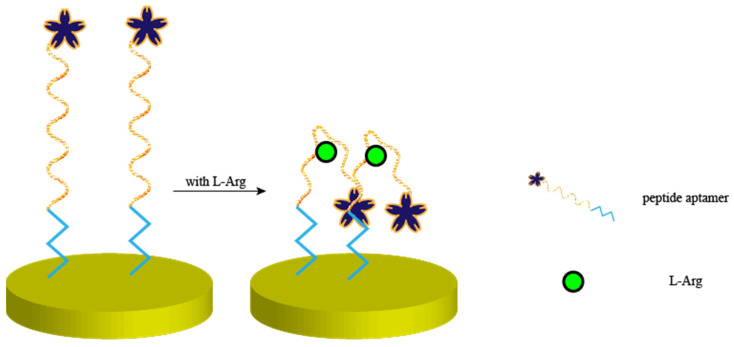
Peptide-based electrochemical sensors for the detection of L-Arg. Zhu et al. constructed a peptide-based electrochemical sensor that, when arginine binds to the peptide, causes the peptide conformation to bend and the peptide-terminal signaling molecules to approach the electrode interface, which results in an increase in the induced current.

Zhu et al. [27] fabricated an electrochemical sensor for the precise recognition of arginine (L-Arg) using a peptide aptamer with a terminal modification of ferrocene, as shown in Figure 2. This modification caused conformational bending when binding to the peptide aptamer in the presence of L-Arg, resulting in an enhanced differential pulse voltammetry (DPV) response signal. The detection limit and range of the sensor were 31 pM and 0.0001–10 μM, respectively. He et al. [28] constructed an impedance-based electrochemical sensor for the detection of L-Arg using the same specific recognition of the L-Arg peptide sequence, in which the surface resistance of the electrode increased with the concentration of L-Arg. The sensor had an extremely wide linear range of 0.1 pM–0.1 mM and a low detection limit of 0.01 pM.

Using electrochemical sensors that are assembled from peptides for the detection of small molecules, such as amino acids, and possibly using them for the detection of amino acids in pig serum without complex pretreatment is the first of its kind. Most studies focused on detecting large molecules, such as proteins or hormones in serum, but there were no previous studies confirming that peptides can specifically recognize small molecules, such as amino acids. Moreover, the authors [28] used a computer to simulate the binding of peptides to amino acids and then used isothermal titration calorimetry to identify the resulting peptides for practical validation. The specificity of the constructed electrochemical biosensor and its performance in real serum assays were excellent following conventional Au–S bond immobilization.

Baek et al. [29] compared the binding forces of eight peptides that can recognize human norovirus to identify the most sensitive peptide and built an impedance electrochemical sensor. The sensor had a detection range and limit of 10–10^5^ and 2.47 copies/mL, respectively.

Cho et al. [30] constructed a peptide-based electrochemical sensor for the detection of neutrophil gelatinase-associated lipocalin (NGAL). The authors screened peptides that specifically bound to NGAL, modified them differently and compared the performance of the sensor with five modifications. NGAL BP1 was selected as the sensor recognition probe. The detection range and limit of square wave voltammetry (SWV) were 0.0001–7.5 μg/mL and 3.93 ng/mL, respectively.

Alvarez-Martos et al. [31] developed an RNA aptamer electrochemical sensor to directly detect dopamine concentrations in animal serum. The authors linked alkanethiol to a half-bladder at the end of the RNA single strand and immobilized the aptamer on the surface of an Au electrode using Au–S bonds. After assaying the dopamine concentration in the serum, the sensor was immersed in PBS for 10 min, which eliminated the interference caused by adsorbed proteins and cells in the serum on the electrode surface. The sensor had a detection range and limit of 0.1–1 μM and 67 nM, respectively.

Konari et al. [32] constructed an electrochemical sensor based on a DNA aptamer to detect thrombin content in cerebrospinal fluid and serum samples from healthy humans and patients with diseases. The aptamer was immobilized on top of layered double hydroxides (LDH), and owing to the poor electron transfer ability of LDH, carbon nanotubes and LDH were co-laminated with the working electrode surface to increase the conductivity of the laminate layer and reduce the resistance. This method of fixing the aptamer improved the stability of the aptamer sensor compared with other methods. Its detection limit and range were 0.1 fM and 0.005–12,000 pM, respectively.

Tavakkoli et al. [33] fabricated a current-based electrochemical sensor based on nucleic acid aptamers for cocaine detection. Ascorbic acid was used to reduce the surface of the Au electrode to obtain a nanoporous Au (NPG) electrode. Cocaine aptamers with modified disulfide bonds were then immobilized on the NPG surface to achieve the sensitive detection of cocaine. The other end of the aptamer was modified with 2, 5-dihydroxybenzoic acid (DHBA). The apta-sensor presented two linear responses in the concentration ranges of 0.05–1 and 1–35 mM and exhibited an excellent detection limit of 21 nM.

Li et al. [34] constructed an aptamer- and current-based biosensor for detecting 5-hydroxytryptamine (5-HT) in human serum. The authors first enclosed positively charged poly(diallyldimethylammonium)-wrapped oxidized single-walled carbon nanotubes (PDDA-oSWCNTs) onto the surface of a screen-printed electrode modified with Au nanomaterials. Subsequently, they immobilized negatively charged 5-HT aptamers onto the PDDA-oSWCNTs. The aptamer was then immobilized on top of the PDDA-oSWCNTs, and tyrosinase was overlaid on top of the aptamer to enhance the selectivity of the sensor. The detection range of the sensor was divided into two segments, from 0.05 to 0.5 and 1 to 20 μM, with a detection limit of 2 nM.

Bagheri Hashkavayi and Raoof [35] assembled a double-stranded DNA-based electrochemical sensor for tryptophan detection. A DNA strand-end-linked thiol was immobilized on the electrode by first covering the screen-printed electrode surface with multiwalled carbon nanotubes and chitosan, followed by the electrodeposition of Au NPs. The DNA strand that complements and specifically recognizes tryptophan was linked to the previous DNA strand via hydrogen bonding. Subsequently, [Fe(bpy)_3_] (p-CH_3_C_6_H_4_SO_2_)_2_ was inserted as a signal molecule and mosaicked onto the double-stranded DNA. When tryptophan was added to the solution, the double-stranded chain unraveled because the specific recognition DNA aptamer bound more strongly to tryptophan, resulting in a decrease in the electrode surface resistance and an increase in the current. The signal molecule was released, and the DPV response current decreased as a basis for detecting tryptophan. The detection limit and range of the electrochemical sensor based on the double-stranded DNA aptamer were 1.00 nM and 3.00–100,000.00 nM, respectively.

All of the above electrochemical sensors based on nucleic acid aptamers are suitable for the rapid detection of components in body fluids, providing new solutions for the development of wearable or implantable devices for real-time monitoring of human metabolites [36,37,38]. For example, the graphene field-effect transistor-based electrochemical aptamer sensor designed by Wu et al. can be used for real-time monitoring of dopamine [39]. The sensor can be directly implanted into the mouse brain while having near cell-scale spatial resolution. The sensor detection limit is 10 pM. Reynoso et al. designed aptamer-based electrochemical sensors that are wearable patches fabricated using microneedle sensor arrays [40]. The sensor can be directly affixed to the skin surface of mice for real-time monitoring of interstitial dermal fluid. Fernández-Vega et al. presented the first implantable aptamer-based platinum microelectrochemical sensor for continuous measurement of the non-electrically active molecule neuropeptide Y [41]. The sensor has a detection limit of 1 pg/mL. The agarose hydrogel protective layer-modified electrochemical aptamer sensor designed by Li et al. can be used to monitor kanamycin in real time in a complex, dynamically changing environment in vivo [38]. Zhao et al. also used an aptamer-modified field-effect transistor approach to construct electrochemical biosensors that can be used for the detection of serotonin in cerebrospinal fluid [42]. The authors developed a high-throughput process for the fabrication of thin and flexible polyimide probes, enabling minimally invasive implantation with detection limits down to the fM level.

Meanwhile, aptamer sensors have been developed that can detect multiple metabolites simultaneously [43]. For example, Wang et al. designed an electrochemical sensor based on a tetrahedral rigid structural base, which enhances the stability of the aptamer [44]. The sensor can simultaneously detect proteins, ions, biomolecules, and SARS-CoV-2. Especially for SARS-CoV-2, the detection limit of the sensor reaches ~0.02 copies per microliter. Gao et al. designed a graphene field-effect transistor-based aptamer sensor for real-time rapid detection of serotonin and dopamine in cerebrospinal fluid [45]. The detection limit of the sensor for both substances was 10 pM. Also, an electrochemical aptamer sensor based on graphene field-effect transistor was designed by Wu et al. [46]. Although the sensor was only used for the detection of E. coli, the authors theoretically discussed the effect of E. coli attachment on the carrier density of probe-modified graphene and experimentally verified that the detection limit of the sensor was 100 CFU/mL. These findings provide a theoretical basis for a wider range of aptamer sensors of the same type for bacterial detection.

### 2.2. Colorimetric or Fluorescent Sensors

**Figure 3:**
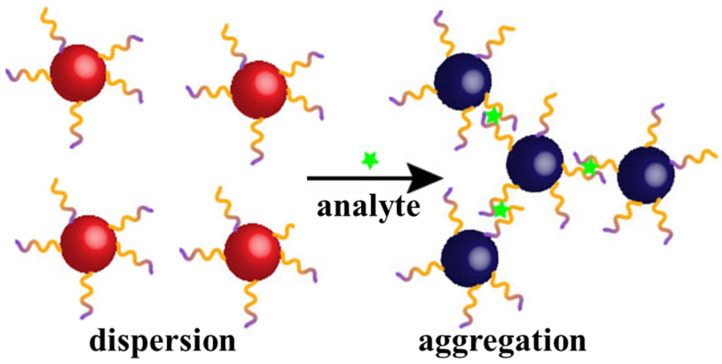
Detection mechanism of colorimetric sensors based on aptamer-modified Au nanoparticles. The aptamer is modified to the surface of the nanogold by gold–sulfur bonding, and when the analyte is present, the aptamer binds to the analyte, leading to aggregation of the nanogold, which causes the colloid color to change from red to blue.

Aptamer-based colorimetric sensors typically use Au nanomaterials as substrates. A nanogold solution is used to detect the analyte because of its different colors in the dispersed and aggregated states. In contrast to conventional electrochemical sensors, colorimetric sensors are gaining increasing attention because of their rapid feedback as well as their ease of operation and readout. Typically, an aptamer is linked to the surface of the Au nanomaterial via an Au–S bond. When an analyte is present in the solution, the aptamer and the analyte are attracted to each other, thus causing aggregation of the Au nanomaterial. Figure 3 illustrates the detection mechanism of colorimetric sensors based on aptamer-modified Au nanoparticles.

In contrast, fluorescent sensors typically use two groups to modify the aptamer: fluorescent and quenching groups. When an analyte is present in the solution, the aptamers are attracted to each other via the analyte, thereby quenching the fluorescence for detection.

**Figure 4:**
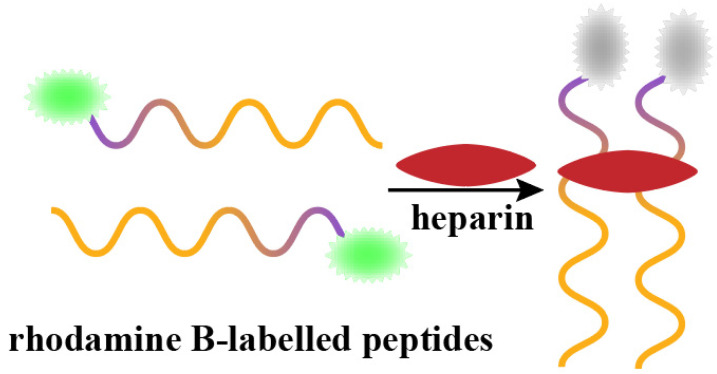
The colorimetric scheme designed by Guo et al. was used for the detection of heparin, which causes the fluorescent groups at the ends of peptides to come into close proximity to each other, thereby quenching their fluorescence.

Guo et al. [47] assembled a sensing platform to detect the heparin levels in human serum. The peptide-based fluorescent sensor for heparin detection is illustrated in Figure 4. The C-terminus of the peptide chain was linked to rhodamine B fluorescein, which was used as a signal source for heparin detection. The fluorescence intensity of rhodamine B reached its maximum at 550 nm. When heparin was added to phosphate-buffered saline (PBS), a specific recognition of the heparin polypeptide chain bound to heparin; this caused the polypeptide conformation to bend and brought the two fluorescent groups in close proximity, thus quenching the fluorescence. The fluorescence intensity was inversely proportional to the heparin concentration. For example, the higher the heparin concentration, the lower the fluorescence intensity. The sensor achieved good linearity in the ranges of 0.01–0.1 and 1.0–70.0 nM, with a detection limit of 0.075 nM.

Tang et al. [48] constructed a fluorescent luminescence sensor for lipopolysaccharide (LPS) detection, which could be directly recognised by the human eye. This sensor can also be used to remove LPS from tissue fluids. Linking of the 1-(4-carboxylbenzene)-1,2,2-triphenyl (CTPY) fluorescent group to the end of the hexadecapeptide served as a signal for LPS detection. When the CTPY-peptide and LPS were present in the solution, LPS bound to the peptide to restrict the intramolecular rotation of the CTPY-peptide. The energy was emitted as radiation, which excited blue light at 340 nm. Simultaneously, the hexadecapeptide aptamer was immobilized onto the surface of mesoporous silica nanospheres, which adsorbed LPS from the solution, thus decreasing the blue light. The detection limit and range of the sensor were 6.97 nM and 0.1–1 μM, respectively.

Parnsubsakul et al. [49] assembled a colorimetric sensor based on amphoteric polypeptides to detect Ni^2+^ in actual samples. The authors modified two types of amino acids with positive and negative charges on the surface of Au nanoparticles (NPs). When Ni^2+^ was present in the solution, Ni ions triggered the accumulation of Au NPs, resulting in a color change in the solution. The sensor detection range and limit were 60–160 and 30 nM, respectively.

Lim et al. [50] fabricated a peptide-based fluorescent sensor to detect LPS in clinical medicine. The authors linked tetramethylrhodamine (TMRho) to the end of the peptide as a fluorescent light source. Subsequently, the peptide was adsorbed onto the surface of graphene oxide (GO), where the fluorescent group was quenched by the influence of GO. When LPS was present in the solution, the stronger binding of LPS to the peptide caused the peptide to move away from GO, thus resuming the fluorescence. The sensor detection range and limit were 0–20 nM and 130 pM, respectively.

Gong et al. [51] constructed a peptide-based colorimetric sensor to detect aldehydes in the air using a self-assembled Lys-rich peptide chain dispersed on nanofibers (NFs) to form a colored film. When aldehydes were present in the air, the specific binding of the aldehyde to the Lys moiety resulted in a change in the structure of the matrix, which caused a change in the color of the colored film. The sensor detected aldehydes when their concentration in air exceeded 300 ppm.

Lee et al. [52] created a peptide-based colorimetric sensor for detecting *A. niger* spores. The authors screened peptides that specifically bound to *A. niger* spores and combined them with nanogold materials. When *A. niger* spores were present in the solution, the color of the solution changed because the binding of *A. niger* spores to the peptide induced the aggregation of the nanogold material. The sensor had a detection range and limit of 0–1.5 × 10^3^ and 50 spores/mL, respectively.

Mu et al. [53] constructed a peptide and nucleic acid aptamer-based dual-emission fluorescent sensor for detecting vancomycin (Van) in serum. The authors creatively proposed the use of a dual aptamer of peptides and single-stranded DNA as a recognition probe by modifying the two different aptamers with different fluorescent light sources. Peptide-linked aggregation-induced emission luminogens and DNA strand-modified gold nanoclusters, with maximum absorption wavelengths of 470 and 650 nm, respectively, were used for the ultrasensitive detection of Van. The detection range and limit of the sensors were 0.01–100 mg/mL and 2.79 ng/mL, respectively.

A double-stranded DNA-based fluorescent sensor was assembled by Geng et al. [54] for the detection of Pb^2+^ in clinical samples. First, when Pb^2+^ was not present in the solution, the fluorescein AMT and complementary double-stranded DNA did not interfere with each other, and the AMT fluoresced normally. However, when Pb^2+^ was present in the solution, the aptamer bound more strongly to Pb^2+^. This phenomenon caused the double strand to unwind, with one single strand of DNA binding to Pb^2+^ and the other single strand changing its conformation to a G-quadruplex and binding to AMT, thereby quenching the fluorescence. The sensor had a detection range and limit of 0.1–1.0 μM and 3.6 nM, respectively.

Xia et al. [55] constructed a double-stranded DNA fluorescence sensor based on modified fluorescent groups at both ends to detect aflatoxin B_1_ (AFB_1_) in peanut oil. The authors modified both ends of aptamer-recognizable AFB_1_ with fluorescent groups, as well as placing quenching groups at both ends of the complementary strand. When AFB_1_ was absent in the solution, the fluorescence was quenched due to the binding of the two single chains, resulting in a fluorescent group close to the quenching group. When AFB_1_ was present in the solution, AFB_1_ bound to the aptamer, thus releasing the fluorescent group and causing it to fluoresce again. The sensor had a detection range and limit of 1–200 and 0.91 ng/mL, respectively.

Aptamer-based colorimetric sensors will provide a cheaper and more convenient solution for real-life health monitoring due to their fast color development, high specificity, and easy operation [56]. For example, the peptide aptamer-based colloidal gold rapid test solution for tryptophan [57] can be used for the rapid screening of health problems, such as insomnia, depression, and mental illness. It can also be used for rapid qualitative screening of hazardous residues in food, such as pesticide residues, veterinary drug residues, and heavy metal ions. Kim et al. designed a tyrosine-based fluorescent sensor for the rapid detection of mercury ions in aqueous solutions and living cells [58]. The detection limit was 33 μg/L. A peptide-based fluorescent probe designed by Lee et al. can be used for the rapid detection of lead ions in aqueous solutions and human serum with a detection limit of 3.8 nM [59]. Using an aptamer-peptide coupling as a recognition probe, Peng et al. constructed a fluorescent sensor that can be used for the rapid detection of lead ions in food with a detection limit of 12.45 nM [60]. Lu et al. designed a peptide-based colorimetric sensing strategy for the rapid detection of lead ions in aqueous solutions [61]. The authors also developed a circuit system based on the concentration of lead ions, which is based on the concentration of lead ions and, thus, realizes the dimming and extinguishing control of the lamp.

Meanwhile, multi-aptamer-modified fluorescent sensors for multi-substance detection are also a key area of research nowadays. Poudineh et al. designed a fluorescent aptamer immunoassay bead based on a “sandwich” type of bead that can simultaneously detect multiple analytes (e.g., glucose and insulin) in blood [62]. In addition, although the arginine sensor designed by Wu et al. can only detect a single amino acid, the authors creatively combined electrochemical and colorimetric methods to achieve rapid detection by naked eye colorimetry and precise quantification by electrochemistry [63]. Xiao et al. synthesized a peptide in-house using solid-phase synthesis, which can be used for the simultaneous detection of copper ions and histidine [64]. The sensors did not interfere with each other, and the detection limits were 0.29 μM and 15.3 nM for the copper ions and histidine, respectively. Zheng et al. used 5-FAM as a fluorescent moiety to modify the peptide and synthesized a fluorescent probe for the rapid detection of copper and sulfur ions [65]. Furthermore, the authors immersed test strips in the fluorescent probe solution, thus creating a portable visual detection method. The detection limits of the colorimetric sensor for the two were 0.48 μM and 1.22 μM, respectively.

### 2.3. Electroluminescent Sensors

Electroluminescent sensors typically use g-C_3_N_4_ or Ru(bpy)_3_^2+^ as the light source. The detection mechanism of some electroluminescent sensors is similar to that of the electrochemical sensors described above, in that the binding of the analyte to the aptamer results in the enhancement or attenuation of the electroluminescent signal. In this section, we focus on a novel mechanism, namely the “shear” mechanism.

**Figure 5:**
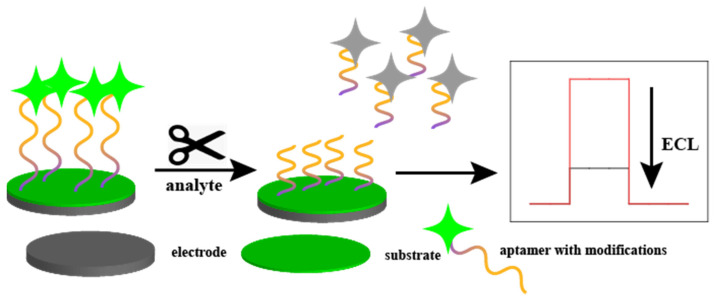
Aptamer-based detection mechanism for electroluminescent sensors. The analyte acts as a pair of scissors that shears the aptamer when the analyte binds to the aptamer, causing the signaling molecules at the end of the aptamer to detach from the electrode interface and lose fluorescence.

Luminol fluorescent materials are usually modified at the end of the aptamer, and the analyte in solution corresponds to the “shear”. When the aptamer is clipped, the interruption of the current results in the attenuation of the fluorescence signal, which is used as the detection signal. Figure 5 illustrates an aptamer-based detection mechanism for electroluminescent sensors.

Xu et al. [66] fabricated a peptide aptamer-based electroluminescent sensor. First, a layer of the conducting polymer, poly(3,4-ethylenedioxythiophene) (PEDOT), was electrodeposited on the surface of the glassy carbon electrode to enhance its conductivity and increase its specific surface area. Next, Au@luminol NPs were incubated, and the peptide was immobilized on the electrode surface using a sulfhydryl group at the N-terminus of the peptide to bond with Au NPs. Finally, polyamidoamine-quantum dots (PAMAM-QDs) were immobilized on the C-terminus of the peptide. In the absence of thrombin, the quantum dots at the end of the electrode fluoresced under voltage excitation. When thrombin was added to the solution, it functioned as a “pair of scissors” and cut the peptide short, causing the PAMAM-QDs to lose voltage and quench the fluorescence. A significant decrease in fluorescence intensity was detected using an ECL workstation. The detection limit and range of the peptide-based sensor were 1.82 fM and 10.0 fM–1.0 nM, respectively.

Fan et al. [67] constructed an electrochemical luminescence sensor based on a peptide sandwich structure to detect LPS. Peptides that recognised LPS were modified differently. One part of the peptide was fixed to the surface of the Au electrode, and the other part was linked to the end of Ru_1_@SiO_2_. When LPS was present in the solution, it was first attracted by the polypeptide and adsorbed to the Au electrode. Subsequently, the polypeptide modified with luminescent material was attracted by LPS and adsorbed to the Au electrode, thus stimulating fluorescence. The detection range and limit of the sensor were 1.0–500 and 0.3 ng/mL, respectively.

Gu et al. [68] created an electrochemical luminescence sensor based on ring polypeptides for glucose detection. Au NPs were reduced on g-C_3_N_4_ to obtain AuNPs/g-C_3_N_4_ composites as electro-chemiluminescent materials. Subsequently, the ring peptide was fixed on a nanogold material to detect glucose. The detection range and limit of the sensor were 1–100 mmol/L and 0.57 nmol/L, respectively.

Fan et al. [69] fabricated a polypeptide-based electrochemiluminescence sensor for matrix metalloproteinase 2 (MMP-2) detection. The authors first combined Ru(bpy)_3_^2+^ with nitrogen-doped graphene quantum dots (NGQDs) bonded via electrostatic attraction. They then doped them with silica nanomaterials to produce an electrochemiluminescent material, which was then attached to the polypeptide terminal. When MMP-2 was present in solution, it functioned as a pair of scissors to cut the peptide, thus moving the luminescent material away from the electrode and weakening the electroluminescence signal. The detection range and limit were 0.01–185 ng/mL and 6.5 pg/mL, respectively.

Various methods to obtain a signal output from peptide-based biosensors can be employed. These include the fluorescent or colorimetric sensors (where the peptide chain is directly attached to a fluorescein surface) or the current or impedance-based electrochemical sensors (where the peptide is immobilized on an electrode surface by means of an Au–S bond). In addition, electroluminescent sensors are obtained by linking quantum dots to the end of the peptide. The peptide can also be bound to an analyte in various ways. For example, binding the peptide to an analyte causes a change in peptide conformation, and using the analyte as “scissors” to cut the peptide can cause a change in signal.

The existing colorimetric sensors primarily use Au nanomaterials. These materials can be rapidly and easily synthesized because of their significant color changes in dispersed and aggregated solutions. They are often linked directly to peptides with specific recognition functions and cause the aggregation of Au nanomaterials via the binding of the peptide to the analyte, thus causing a color change in the solution.

Yang et al. [70] constructed an aptamer-based electrochemiluminescence sensor for the detection of okadaic acid (OA). The authors dissolved electroluminescent Ru(bpy)_3_^2+^ in a homogeneous solution rather than on a narrow electrode surface. The authors first adsorbed the aptamer containing Ru(bpy)_3_^2+^ onto the surface of magnetic GO (M-GO). When OA was present in the solution, the aptamer bound to OA, resulting in the release of the aptamer from the M-GO surface. Ru(bpy)_3_^2+^ further dissociated in the presence of deoxyribonuclease I, causing fluorescence. The sensor had a detection range of 0.01–10.0 ng/mL and a detection limit of 4 pg/mL.

Li et al. [71] fabricated an aptamer-based dual-fluorescent signal electrochemiluminescence sensor to detect DNA methyltransferase (MTase) activity. The authors first incubated an antipollution peptide onto the surface of a nanogold material. Subsequently, a specific hairpin aptamer was linked to the end of the peptide, and the other end of the aptamer was modified with the fluorescent probe Au@luminol. When MTase was present in the solution, it linked the aptamer, and the residual portion was amplified via a hybridization chain reaction and extended into a DNA double strand. Subsequently, PTC–NH_2_ in solution was inserted into the DNA double strand via adsorption of the aptamer, triggering a fluorescent signal. The ratio of Au@luminol at the end of the hairpin aptamer, which was not sheared by MTase on the electrode surface, to the fluorescent signal of PTC–NH_2_, was linearly correlated with the concentration of MTase. The sensor had a detection range and limit of 0.05–100 and 0.02 U/mL, respectively.

Khonsari and Sun [72] constructed an aptamer-based electroluminescent sensor to detect lysozyme bacteria in human serum. The authors used Au nanomaterials as a substrate to immobilize the aptamer and then attached NGQDs and persulphate (NGQD-S_2_O_8_^2−^) to the end of the aptamer for the detection of lysozyme bacteria. The sensor had a detection range from 10 fM to 10 nM and a detection limit of 0.8 fM.

Sha et al. [73] assembled an aptamer-based electroluminescent sensor for cytochrome c (Cyt C) detection. The authors used Ru(bpy)_3_^2+^-doped silica NPs (RuSiO_2_ NPs) as the electroluminescent material with modified ferrocene at the end of the aptamer. When Cyt C was present in the solution, the aptamer was attracted to Cyt C and adsorbed onto the electrode surface. Because the aptamer with ferrocene modified at the end quenched the fluorescence of the RuSiO_2_ NPs, it was used for detection. The sensor detection range was 0.001–100 nM, with a detection limit of 0.48 pM.

Lin et al. [74] constructed an aptamer-based electroluminescent sensor for the detection of thrombin. The authors synthesized flower-like titanium dioxide (f1-TiO_2_) nanomaterials. By modifying 2D graphite-like carbon nitride (g-C_3_N_4_) in f1-TiO_2_, the capacity of g-C_3_N_4_ was enhanced while facilitating the action of co-reactants. Subsequently, a specific aptamer terminally modified with ferrocene was attached to the surface of g-C_3_N_4_, which resulted in a weakened fluorescence signal due to the quenching of the fluorescence of g-C_3_N_4_ by ferrocene. However, thrombin binds to the aptamer when present in the solution, resulting in the aptamer releasing from the g-C_3_N_4_ surface, which again excites fluorescence. The sensor had a detection range from 10^−11^ to 10^−5^ M and a detection limit of 8.9 × 10^−12^ M.

Compared to the first two sensors, aptamer-based electroluminescent sensors have fewer application scenarios. The need for both a power source as well as a fluorescence detector requires supporting proprietary equipment, thus limiting their application and development. However, with the advancement of science and technology, the advantages of electroluminescent sensors will be further explored, and their application potential will be very broad. In Table 1, we summarize the aptamer-based sensors constructed by predecessors and compare their performance.

### 2.4. Peptides as an Antipollution Layer

Peptides can also be used as modifying materials to assemble biosensors, especially as antifouling layers. Several groups of researchers have found that the pairing of an acidic amino acid (L-Glutamic acid) and a basic amino acid (L-Lysine) is effective in resisting interference caused by the adsorption of irrelevant proteins in serum [75,76]. Meanwhile, the D-amino acid also has the same function, as well as even better antifouling effect than the same L-amino acid [77,78]. This may be due to the electrically repulsive nature of the charged amino acids and proteins. Usually, peptides as modifying layers can be divided into two categories. Peptides in the first category, namely site sealers, act similarly to mercaptohexanol. After the electrode interface has been incubated with the aptamer, the unbound site is closed using a peptide with antifouling properties [79]. The second type of peptides are modified directly on the end of the aptamer, and this method simplifies the sensor assembly process. Compared to anti-fouling materials, such as mercaptohexanol and polyethylene glycol, peptides have better biocompatibility and safety [80,81].

Peptide monolayers are synthetically simple to prepare and highly tunable with the broad range of available natural and synthetic amino acids [82]. The binding affinity of genetically engineered peptides for inorganics is derived from the sum of the weak electrostatic and van der Waals interactions between the peptide and solid surfaces, requiring no complex or aggressive chemistries [83].

**Figure 6:**
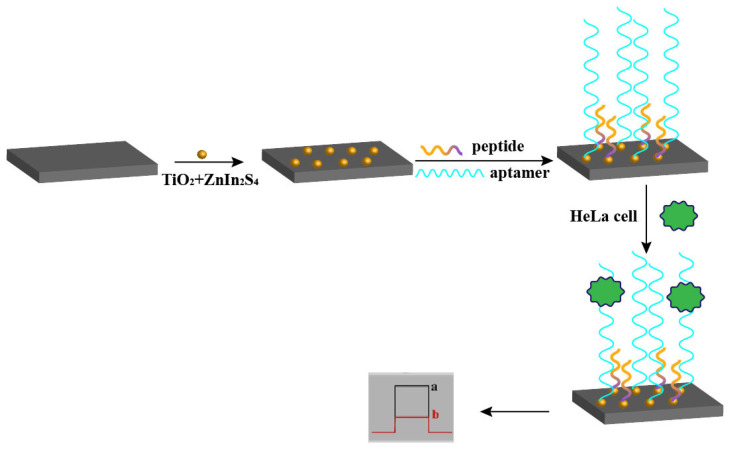
Aptamer-based electroluminescent sensor using peptides as the antifouling layer for the detection of MCF-7 cells. Liu et al. designed an aptamer-based electroluminescent sensor for the detection of MCF-7 cells. When MCF-7 cells were bound to the aptamer, it resulted in a decrease in the interfacial current and a weakening of the light signal.

Liu et al. [84] constructed a nucleic acid aptamer-based electrochemical sensor to detect human breast cancer cell lines (MCF-7 cells) in human serum. Figure 6 illustrates the aptamer-based electroluminescent sensor using peptides as the antifouling layer for the detection of MCF-7 cells. The authors used a polypeptide chain with branching as the antipollution layer and polyaniline as the substrate material to prepare a biosensor with a significantly better antipollution capability than the conventional PEG antipollution layer and single-chain linear polypeptide. CPPPPEK2(EK)4(EK) was used as the branching polypeptide chain. The detection range and limit of the biosensor were 50–10^6^ and 20 cells/mL, respectively.

Qian et al. [85] developed a fiberoptic surface plasmon resonance biosensing technique for detecting platelet-derived growth factors in human serum using nucleic acid aptamer-modified nanogold-enhanced signals. The authors also used an amphoteric peptide as the antifouling layer with the peptide sequence EKEKEKE-PPPPC, except that the peptide was immobilized directly onto the fiberoptic surface together with the nucleic acid aptamer instead of onto the conventional bilayer structure (peptide–nucleic acid aptamer). The sensor had a detection range and limit of 1–1000 and 0.35 pM, respectively.

**Figure 7:**
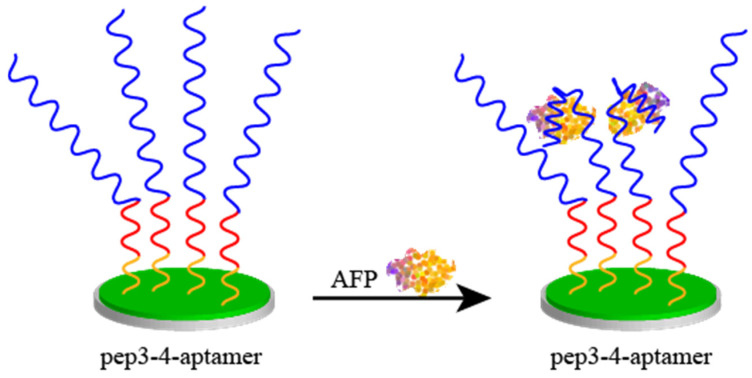
Bilayer peptide-based electrochemical sensor for the detection of alpha fetoprotein (AFP). Recognition of peptide end modifications against contaminating peptides leads to a decrease in current when AFP binds to the recognition peptide.

Liu et al. [86] fabricated a bilayer amphiphilic peptide-based aptamer sensor for alpha fetoprotein (AFP) detection in biological fluids. Figure 7 illustrates the bilayer peptide-based electrochemical sensor for the detection of alpha fetoprotein (AFP). The sensor enhanced the interfacial electron transfer capability by electrodepositing polyaniline on the electrode surface and could be simultaneously used for peptide immobilization. The antifouling peptide layer was immobilized on the electrode surface by linking the amino group of anilines to the C-terminus of the peptide via a peptide bond. The two peptide chains with the best antifouling performance (pep 3-4) were obtained by comparing the four peptide chains. The peptide sequences of pep 3-4 were CPPPPEKEKEKEK and CPPPPEKEKEKEK. The methemoglobin nucleic acid aptamer was then incubated at the end of the polypeptide. Thus, methemoglobin can be specifically detected.

Li et al. [87] developed a nucleic acid aptamer-based electrochemical sensor to detect adenosine triphosphate (ATP). The authors used the first novel composite material, GO and poly(3,4-ethylenedioxythiophene) (GO-PEDOT), to cover the surface of a glassy carbon electrode. An antipollution peptide with the sequence EKEKEKE was then immobilized onto the membrane surface, and an ATP aptamer was linked to the end of the peptide, thus enabling ATP detection. The detection range and limit of the sensor were 0.1 pM–1.0 μM and 0.03 pM, respectively.

Li et al. [88] constructed a nucleic acid aptamer-based electrochemical sensor for detecting prostate-specific antigens (PSAs). The authors used a conventional coating material, polyaniline, in combination with nanogold for peptide immobilisation. The antipollution peptide sequence, CRERERE, was then immobilised on the surface of the nanogold material and linked to the PSA aptamer. The constructed sensor had a detection range from 0.1 pg/mL to 100 ng/mL and a detection limit of 0.085 pg/mL.

Fan et al. [89] fabricated an aptamer-based photoelectrochemical cytosensor for the detection of cervical carcinoma Henrietta Lacks (HeLa) cells in biological fluids. The authors used TiO_2_ NPs and ZnIn_2_S_4_ nanocrystals as photoelectrochemical sensor substrates. Moreover, an antipollution peptide layer and an aptamer were immobilized on the surface of the nanomaterials. When the aptamer was bound to HeLa cells, the photoelectric signal was significantly reduced, resulting in the sensitive detection of HeLa cells. The peptide sequence was EESKSESKSGGGGC. The detection range of the sensor was 1.0 × 10^2^–1.0 × 10^6^ cells/mL, with a detection limit of 34 cells/mL.

Hao et al. [90] constructed a dual-fluorescent probe-based ratiometric electrochemiluminescence sensor for the detection of carcinoembryonic antigens (CEA) in human serum. The authors first immobilized an antipollution peptide with CdS quantum dots (CdS QDs), modified at the ends, onto the surface of an indium tin oxide electrode and then covered the peptide with complementary DNA duplexes. One of the duplexes was modified with Au–luminol at the end. When CEA was present, owing to the stronger binding of CEA to the aptamer, the DNA double-strand unraveled, Au–luminol fluorescence was quenched, and CdS QDs fluorescence predominated. This resulted in the sensitive detection of CEA. The polypeptide sequence was DKDKDKDPPPPC. The sensor had a detection range and limit of 0–100 ng/mL and 0.13 pg/mL, respectively.

Researchers typically use acidic (E, glutamic acid; D, aspartic acid) and basic amino acids (K, lysine; R, arginine) in alternating arrangements to synthesize peptides with antifouling abilities. The peptide sequence typically comprises six or eight amino acid residues. This is because amphiphilic peptides made up of this alternating arrangement of positively and negatively charged amino acids can bind more water molecules to form a hydration layer, thus achieving effective resistance to non-specific proteins. Interfacial hydration is considered an important factor that influences the antifouling properties of hydrophilic non-ionic materials. In contrast, Fan et al. reported that inserting hydrophilic amino acids (S, serine; G, glycine) into amphiphilic peptides enhanced their hydrophilicity, thereby preventing the proximity of biofouling molecules to the electrodes via hydrophobic interactions.

Simultaneously, the structure and chain length of amphiphilic peptides can be controlled by adjusting the composition of the peptide sequence, which is an advantage compared to conventional organic polymers. Amphoteric ionic peptides are currently used in functionalized biomedical implants and human epidermal biosensors.

## 3. Conclusion and Future Perspective

### 3.1. Discussion and Conclusions

This paper reviews aptamer-based biosensors in which peptide aptamers, nucleic acid aptamers, and peptides constructed as antifouling layers for sensors are presented. The intersection of aptamers and traditional nanomaterials provides a new chapter in biosensor development. Although aptamers provide good biocompatibility, high specificity, and extremely low detection limits, nanomaterials with good physical and chemical properties can increase the sensitivity of sensors.

After investigating the literature on aptamer-based biosensors mentioned above, we believe that although electrochemical-based aptamer sensors have an extremely wide detection range and a very low detection limit, their stability and reproducibility still need to be improved. For example, aptamers behave differently in different buffers (e.g., PBS, EDTA buffer, tirs buffer, etc.), which may be due to the different conformational stretching of the aptamer in buffer systems with different ions, and the effect of conformation on the affinity is very critical. At the same time, since the aptamer itself is charged, the question of whether the conformation as well as the viability of the aptamer is affected in the case of energization is still open to debate.

Meanwhile, although the detection limit of aptamer-based modified colloidal gold solution is higher, its strong specificity, rapid color development, easy operation and commercialization have instead led to the aptamer-based colloidal gold gaining more attention. For example, aptamer-based colloidal gold solutions for the rapid detection of heavy metal ions or amino acids [57,91,92,93,94,95] are expected to be industrialized for the safety and nutritional value assessment of seafood or food. These new technologies will hopefully replace the currently available colloidal gold chromatography based on antibody–antigen reactions for more accurate and cheaper rapid testing.

Of course, we also introduce some of the fluorescent probe-modified aptamer sensors, which will provide a solid foundation for the in vivo imaging of animals. Due to the good biocompatibility of the aptamers, the fluorescent probe-modified aptamers will not be attacked by the immune system when they are delivered into the living body [96,97,98,99,100], which will enable prolonged fluorescence imaging. At the same time, due to the strong specificity of the aptamer, targeted imaging of the fluorescent probe can be realized. These studies will provide easier tools for in vivo metabolic amino acid studies and can also be used for targeted imaging of viruses or tumor cells for biomedical as well as clinical medicine.

### 3.2. Future Perspective

Notably, while wearable and implantable sensors have gained tremendous momentum, few devices are clinically applicable. Although several of the current aptamer-based sensors, such as electrochemical, optical, and magneto-fluidic sensors, have outstanding sensitivity and specificity, they still require time-consuming preprocessing, such as labelling and pre-analytical assays, when confronted with real samples. In certain cases, labelling methods are not suitable because the labelled material may occupy important binding sites or cause spatial resistance, thereby affecting the detection results.

The introduction of flexible hydrophilic materials has resulted in the development of wearable sensors. This field has a promising future. Wearable sensors enable the non-invasive and real-time monitoring of devices and can play a role in the early detection of diseases by analyzing the content of body fluid components. Electrochemical sensors offer rapid, simple, and sensitive methods for the detection of cancer and Alzheimer’s and Parkinson’s disease. Therefore, flexible nanomaterial-based biosensors have become the focus of analytical chemistry.

Aptamer-based sensors are currently well studied for the detection of various compounds as well as the use of aptamers as tools for in vivo imaging or targeted delivery. However, most of the current research is still only applicable in the laboratory, i.e., it can only be realized in a more stringent environment, and very few products are actually available for real-life use. Meanwhile, aptamer-based fluorescent probes for targeted in vivo imaging can only be realized in vivo in animals (e.g., mice), but not in broader applications. Enhancing the stability and robustness of aptamer-based sensors to cope with various complex interferences in real life is the focus and difficulty of current research. At the same time, reducing the pre-treatment of real samples, especially meat, vegetables, feed, and seafood, is the current difficulty in realizing rapid detection. On the other hand, although aptamers for targeted imaging have good biocompatibility, they are still subject to the influence of various enzymes in vivo. Therefore, aptamer-based fluorescent probes still require various embedding materials.

Although there are still some urgent problems that need to be solved, it is undeniable that aptamer-based sensors have gained a lot of attention as a new kind of recognition probe and, at the same time, still have an immeasurable potential for development. After solving the above problems, aptamer-based sensors will gain more applications in the field of rapid food testing, biomedicine, and clinical medicine. Aptamer-based sensors will enable faster, more accurate, and cheaper detection, as well as more comfortable and precise treatment.

## Figures and Tables

**Table 1:** Analytical figures of merit of the aptamer-based biosensors.

Probe	Sequence	LOD	Linear Range	Analyte	Method	Ref.
Peptide	HHHHHHHGGGGGENIMPVLGC	0.5 nM	0.25–7.5 nM	UlaG	EIS	[26]
Peptide-Fc	GGGGGFGHIHEGYGGGGK	31 pM	0.0001–10 μM	L-Arg	DPV	[27]
Peptide	GGGGFGHIHEGY	0.1 pM	0.1 pM–0.1 mM	L-Arg	EIS	[28]
Peptide	QHKMHKPHKNTKGGGGSGGGGSC	2.47 copies/mL	10–10^5^ copies/mL	Norovirus	EIS	[29]
Peptide	DRWVARDPASIF	3.93 ng/mL	0.0001–7.5 μg/mL	NGAL	SWV	[30]
Peptide-rhodamine B	RNRHTHLRTRPRK	0.075 nM	0.01–0.1 nM, 1.0–70.0 nM	Heparin	Fluorescence	[47]
CTPY-peptide	GCKPTFRRLKWKYKCG	6.97 nM	0.1–1 μM	LPS	Fluorescence	[48]
Au NPs-peptide	EKEKEKPPPPC	30 nM	60–160 nM	Ni^2+^	Colorimetric	[49]
TMRho-peptide	KKNYSSSISSIHC	130 pM	0–20 nM	LPS	Fluorescence	[50]
NFs-peptide	GGGKKK	300 ppm	-	Aldehydes	Colorimetric	[51]
Au NPs-peptide	FTPHPVGRPHTM	50 spores/mL	0–1.5 × 10^3^ spores/mL	A. niger Spores	Colorimetric	[52]
PAMAM-QDs-peptide	DRDRDRDRSGRPVLG	1.82 fM	10.0 fM–1.0 nM	Thrombin	Electroluminescent	[66]
Peptide- Ru_1_@SiO_2_	CIGKLHSAGK	0.3 ng/mL	1.0–500 ng/mL	LPS	electroluminescent	[67]
Au NPs/g-C_3_N_4_-peptide	cyclo-[−CNDNHCRDNDC−]	0.57 nM	1–100 mM	Glucose	electroluminescent	[68]
Ru(bpy)_3_^2+^ -NGQDs-peptide	CGPLGVRGK	6.5 pg/mL	0.01–185 ng/mL	MMP-2	electroluminescent	[69]
RNA	HO-C_6_-S-S-C_6_-5′-UCU CUG UGU GCG CCA GAG ACA GUG GGG CAG AUA UGG GCC AGC ACA GAA UGA GGC CC-3′	67 nM	0.1–1 μM	Dopamine	CA	[31]
ssDNA	5′-NH_2_-AGT CCG TGG TAG GGC AGG TTG GGG TGA CT-3′	0.1 fM	0.005 pM–12 nM	Thrombin	DPV	[32]
ssDNA-DHBA	5′-AGA CAA GGA AAA TCC TTC AA TGA AGT GGG TCG-3′	21 nM	0.05–1, 1–35 mM	Cocaine	SWV	[33]
ssDNA-Tyr	5′-NH_2_-CTC TCG GGA CGA CTG GTA GGC AGA TAG GGG AAG CTG ATT CGA TGC GTG GGT CGT CCC-3′	2 nM	0.05–0.5, 1–20 μM	5-HT	DPV	[34]
Hemin-ssDNA	5′-AGC ACG TTG GTT AGG TCA GGT TTG GGT TTC GTG C-3′	1.0 nM	3.0–100,000.0 nM	Tryptophan	DPV	[35]
ssDNA-Au NCs	5′-NH_2_-CGA GGG TAC CGC AAT AGT ACT TAT TGT TCG CCT ATT GTG GGT GGG-3′.	2.79 ng/mL	0.01–100 mg/mL	Van	fluorescence	[53]
AMT-ssDNA	5′-GGG TGG GTG GGT GGG T-3′	3.6 nM	0.1–1.0 μM	Pb^2+^	fluorescence	[54]
FAM-ssDNA	5′-GTT GGG CAC GTG TTG TCT CTC TGT GTC TCG TGC CCT TCG CTA GGC CCA CA-3′	0.91 ng/mL	1–200 ng/mL	AFB_1_	fluorescence	[55]
Ru(bpy)_3_^2+^- ssDNA	5′-GGT CAC CAA CAA CAG GGA GCG CTA CGC GAA GGG TCA ATG TGA CGT CAT GCG GAT GTG TGG-3′	4 pg/mL	0.01–10.0 ng/mL	OA	electroluminescent	[70]
Au@luminol- ssDNA	5′-HOOC-TTT TTT GAA GGA GGG GCG ATC TTT TTG ATC TTT TT-(CH_2_)_6_-SH-3′	0.02 U/mL	0.05–100 U/mL	MTase	electroluminescent	[71]
NGQD-S_2_O_8_^2−^- ssDNA	5′-HS-(CH_2_)_6_-ATC TAC GAA TTC ATC AGG GCT AAA GAG TGC AGA GTT ACT TAG-3′	0.8 fM	10 fM–10 nM	Lysozyme	electroluminescent	[72]
Ru(bpy)_3_^2+^-RuSiO_2_-ssDNA	5′-CCG TGT CTG GGG CCG ACC GGC GCA TTG GGT ACG TTG C(CH_2_)_6_-NH_2_-3′	0.48 pM	0.001–100 nM	Cyt C	electroluminescent	[73]
g-C_3_N_4_- ssDNA	5′-HOOC-(CH_2_)_6_-ATT TGG CCA ACC ACA CCA ACC-3′	8.9 × 10^−12^ M	10^−11^–10^−5^ M	Thrombin	electroluminescent	[74]

CA, chronoamperometry; Tyr, tyrosinase; Hemin, [Fe(bpy)_3_] (p-CH_3_C_6_H_4_SO_2_)_2_; AMT, 3-(aminomethyl)-2,5,9-trimethyl-7H-furo[3,2-g]chromen-7-one; FAM, fluorophore 6-carboxyfluorescein.
